# Dynamics of binding ability prediction between spike protein and human ACE2 reveals the adaptive strategy of SARS-CoV-2 in humans

**DOI:** 10.1038/s41598-021-82938-2

**Published:** 2021-02-04

**Authors:** Xia Xue, Jianxiang Shi, Hongen Xu, Yaping Qin, Zengguang Yang, Shuaisheng Feng, Danhua Liu, Liguo Jian, Linlin Hua, Yaohe Wang, Qi Zhang, Xueyong Huang, Xiaoju Zhang, Xinxin Li, Chunguang Chen, Jiancheng Guo, Wenxue Tang, Jianbo Liu

**Affiliations:** 1 Academy of Medical Sciences, Precision Medicine Center of The Second Affiliated Hospital of Zhengzhou University, Zhengzhou University, Zhengzhou, China; 2 The Second Affiliated Hospital of Zhengzhou University, Zhengzhou, China; 3 National Centre for International Research in Cell and Gene Therapy, Academy of Medical Science, Zhengzhou University, Zhengzhou, China; 4 Center for Biomarkers and Biotherapeutics, Barts Cancer Institute, Queen Mary University of London, London, UK; 5 BGI College and Henan Institute of Medical and Pharmaceutical Sciences, Zhengzhou University, Zhengzhou, Henan China; 6 State Key Laboratory of Esophageal Cancer Prevention and Treatment, School of Pharmaceutical Sciences, Zhengzhou University, Zhengzhou, China; 7 Henan Province Center for Disease Control and Prevention, Zhengzhou, China; 8 Henan Provincial People’s Hospital, Zhengzhou, China; 9 Henan Hospital of Infectious Diseases, Zhengzhou, China

**Keywords:** Computational biology and bioinformatics, Viral infection

## Abstract

SARS-CoV-2 (severe acute respiratory syndrome coronavirus 2) is a novel coronavirus causing the COVID-19 pandemic in 2020. High adaptive plasticity on the spike protein of SASR-CoV-2 enables it to transmit across different host species. In the present study, we collected 2092 high-quality genome sequences of SARS-CoV-2 from 160 regions in over 50 countries and reconstructed their phylogeny. We also analyzed the polymorphic interaction between spike protein and human ACE2 (hACE2). Phylogenetic analysis of SARS-CoV-2 suggests that SARS-CoV-2 is probably originated from a recombination event on the spike protein between a bat coronavirus and a pangolin coronavirus that endows it humans infectivity. Compared with other regions in the S gene of SARS-CoV-2, the direct-binding sites of the receptor-binding domain (RBD) is more conserved. We focused on 3,860 amino acid mutations in spike protein RBD (T333-C525) of SARS-CoV-2 and simulated their differential stability and binding affinity to hACE2 (S19-D615). The results indicate no preference for SARS-CoV-2 infectivity on people of different ethnic groups. The variants in the spike protein of SARS-CoV-2 may also be a good indicator demonstrating the transmission route of SARS-CoV-2 from its natural reservoir to human hosts.

## Introduction

Coronavirus is commonly found in nature but infects only mammals and birds^1–3^. Among the characterized 46 species, seven of them are human-susceptible^4,5^. Aside from SARS-CoV and MERS-CoV that cause deadly pneumonia in humans by crossing the species barrier^3,6,7^, SARS-CoV-2 is now causing a global pandemic of respiratory disease (COVID-19) since the first confirmed cases in Wuhan city of China^8^. SARS-CoV-2 has been identified as a novel β-coronavirus in the family of Coronaviridae^2^. Up to date, COVID-19 results in more than 80 million people infected and almost two million deaths worldwide. Compared with SARS-CoV and MERS-CoV, SARS-CoV-2 spreads more rapidly due to its higher human infectivity^8–10^. Revealing the origin of SARS-CoV-2 and its strategy for adapting to human hosts is therefore valuable to control the COVID-19 pandemic and develop effective therapeutics and vaccines.

The genome structure of SARS-CoV-2 is similar to other beta-coronaviruses, which is composed of 5′-replicase ORF1ab-S-envelope(E)-membrane(M)-N-3′ containing numbers of open reading frames (ORFs). The phylogenetic relationship of various coronavirus^11^ shows around 70% genome sequence similarity between SARS-CoV-2 and SARS-CoV^11^. SARS-CoV-2 is more closely related to bat coronavirus RaTG13 particularly in the spike (S) gene^1^. It is noted that although bat coronavirus RaTG13 is highly similar to SARS-CoV-2 especially between genes, they differed significantly in some crucial genomic structures^12^. One of the most notable features is a polybasic (furin) cleavage site insertion (PRRA residue) at the junction between two subunits (S1, S2) of S protein^1,2,12,13^, which is uniquely present in SARS-CoV-2. Although some studies show bats could be the reservoir host for many coronaviruses including SARS-CoV, the natural host of SARS-CoV-2 remains uncertain^4,10,14^. Given the global spread of COVID-19, it draws much attention to reveal the origins of the pandemic event. A detailed evolutionary analysis of SARS-CoV-2 may explain its infectivity and transmissibility in different animal hosts and provide evidence indicating whether this virus evolved naturally or created by manipulated engineering.

Spike glycoprotein on the surface is critical for SARS-CoV-2 entry into the target cells by forming protruding homotrimers to recognize the host cell receptor and subsequently cause membrane fusion^15^. Spike protein includes two linked subunits (S1, S2), while the receptor-binding domain (RBD) on the S1 subunit mediates the binding to the peptidase domain (PD) of angiotensin-converting enzyme 2 (ACE2) in host cells. S2 subunit is responsible for subsequent membrane fusion during viral infection^14,16^. In SARS-CoV, spike protein RBD is the most diverse region in the whole genome, among which six amino acids (Y442, L472, N479, D480, T487, and Y4911) have been confirmed to play a pivotal role for ACE2 receptor binding and further its transmission across species boundary^16^. Similar to SARS-CoV, RBD variants acquired in different hosts have also been observed in SARS-CoV-2^16^. Thus, identifying the spike protein RBD variants affecting its binding to the ACE2 receptor would be necessaryfor understanding the transmission mechanism of SARS-CoV-2 in human cells. Furthermore, recent studies have shown that the capacity of hACE2 binding to spike protein of SARS-CoV-2 determines the transmissibility of this coronavirus across species and in different populations^14,17^.

The present study intends to (1) reconstruct the phylogeny of SARS-CoV-2 strains from different populations at the genomic level, (2) identify the variants on spike protein of SARS-CoV-2 based on the S gene sequences and predict their differential stability and the binding affinities to hACE2*,* (3) explore the potential whether the variants in hACE2 from the different ethnic groups may affect the infectivity of SARS-CoV-2. To this end, we analyzed the variants of *ACE2* in a large cohort including 1000 Chinese people and other ethnic groups to identify the polymorphisms that may influence the binding of hACE2 and spike protein of SARS-CoV-2. Furthermore, we predicted the affinities of spike protein RBD that binds to hACE2 variants to reveal whether those changes would influence individuals’ susceptibility to SARS-CoV-2. Herein, our study partially explains the origin of SARS-CoV-2 based on genomic and multiple key gene sequences, which allows us to elucidate the population risk profiles and also help advance therapeutics such as a rationally designed soluble ACE2 receptor for the management of COVID-19.

## Materials and methods

### Data preparation

Two thousand and ninety-two genome sequences of SARS-CoV-2 from 160 regions including 50 countries were downloaded from GenBank (https://www.ncbi.nlm.nih.gov/genbank/) and GISAID (https://www.gisaid.org/) databases, metadata of all the SARS-CoV-2 was shown in Supplementary Table S1. Ten genomic sequences of SARS-CoV and MERS-CoV (five for each), ten genome sequences of pangolin coronavirus and three bat coronaviruses were collected from GISAID as well. The genome-sequencing of a local donor has been completed in a laboratory from the local CDC, and the relevant data is stored in the local CDC laboratory database for further analysis (unreleased). The whole-genome sequences were aligned with Clustal Omega (V1.2.3)^18^ under the default setting of this program. Then we extracted the sequences of S, E, M, N genes from the genome sequences of all beta coronaviruses in our analysis. SARS-CoV-2 Wuhan-01 (NC_045512) was selected as the reference for gene sequences extraction, after alignment, a custom Python script (Supplementary Script 1) was used to extract sequences. In order to analyze the S gene and S, E, M, N genes, we aligned them individually with Clustal Omega (V1.2.3)^18^ and filtered them by sequences (Supplementary Script 1) containing more than 15 continuous unknown bases (N). Three hundred and twenty-seven non-repetitive sequences of S gene and 469 of S–E–M–N sequences of SARS-CoV-2 were extracted from the filtered genome sequences, respectively.

### Phylogenetic analysis

After sequence alignment, Modelfinder in Iqtree (v1.6.12)^19^ was used to determine the most suitable model for each sequence dataset. We chose GTR + G model for genome sequences and JC + I + G for gene tree construction. The maximum likelihood tree was reconstructed using MEGA v5.2^20^ and Iqtree (v1.6.12)^19^. We performed with 1000 Maximum number of iterations and applied approximate Bayes test on our phylogenetic analysis, bootstrap values under replicating resample 1000 times. Figtree v1.4^21^ was performed on editing and screening the evolutionary tree topology. The calculation of genetic distance (whole genome and individual genes) was carried out by Kimura two factor correction method for nucleic acid level calculation. To avoid the prediction error caused by the selection of outgroups with a far evolutionary relationship, the sequence of MERS-CoV and SARS-CoV were included as outgroups to predict the genetic relationship.

### Variants of the spike protein and hACE2 prediction

In silico mutagenesis of SARS-CoV-2 spike protein receptor-binding domain bound to the ACE2 receptor complex (PDB ID: 6M0J) was used to predict the variable influence on binding affinity and protein stability. The proposed residue sites were substituted to 19 other amino acids and an ensemble of the conformations (the number of conformations was limit to 25) was generated for each mutant by low-mode MD (Molecular Dynamics), the parameters we used including iteration limit 50, RMSD limit 0.25, energy window 10, conformation limit 25, fix residues farther than 4.5, 0 tether sidechains and one tether backbone. MM/GBVI was applied to calculate the binding affinity of each conformation and ACE2 molecules. The force field used for calculation was OPLS-AA, and the implicit solvent was the reaction field (R-Field) model. All calculations were performed in MOE (2019.01) (Molecular Operating Environment) software^22,23^. The structure of ACE2 receptor (PDB ID: 6M17) was used to perform in silico mutagenesis. The genomic variants in the human *ACE2* gene for different populations were downloaded from the gnomAD database (https://gnomad.broadinstitute.org/). The proposed residue sites were substituted to the amino acids that have the reported point mutations according to gnomAD. The parameters used to predict the polymorphism of hACE2 binding to the spike protein of SARS-CoV-2 are the same as the previous parameters for predicting the variants of the spike protein binding to hACE2. The statistical analysis for affinity and stability of the complex was calculated in the MOE, the cut off of the dAffinity refers to being strong affinity was set as definite three while definite one for high stability^24,25^.

## Results

### Phylogeny of SARS-CoV-2

**Figure 1:**
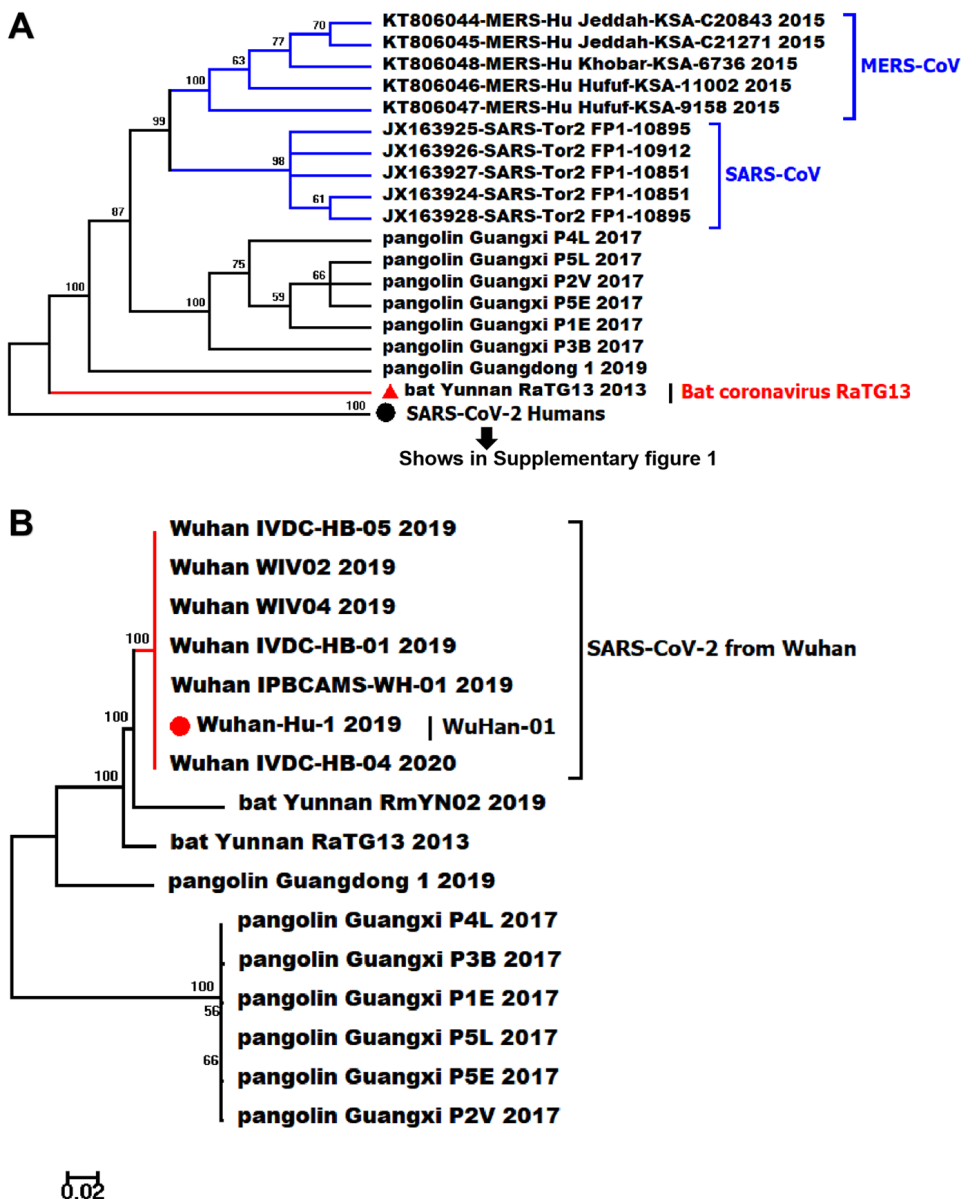
The ML phylogeny tree of different strains SARS-CoV-2 from the various region all around the world based on whole-genome sequences (Partial, complete phylogenetic tree is showen in Supplementary Fig. S1), the bootstrap values were mapped on the branch as long as the colors annotated for all clades. (**A**) The phylogenetic relationship reconstructed with genome sequences of SARS-CoV, MERS-CoV and pangolin/bat coronaviruses, SARS-CoV-2 strains from different regions showed in one consensus branch on the bottom of the sub-tree with bootstrap value 100, SARS-CoV-2 human on the bottom includes branches of SARS-CoV-2 from 164 regions globally (Supplementary Fig. S1); (**B**) The phylogenetic relationship of SARS-CoV-2 collected from Wuhan city of China in the early time of COVID-19 pandemic and beta coronaviruses derived from bat and pangolin.

The Maximum Likelihood (ML) phylogenic trees were constructed based on 2,112 genome sequences of beta coronaviruses derived from different hosts, with SARS-CoV and MERS-CoV selected as the out-groups (Fig. 1a). After alignment, we merged identical sequences into one clade with labels kept as one. The genomic tree showed all SARS-CoV-2 is closely related to the beta coronavirus isolated from the horseshoe bat (RaTG13 and RmYN02), and pangolin coronaviruses were the ancestor that give rise to bat coronaviruses and SARS-CoV-2 in humans. According to sequence alignment, SARS-CoV-2 is not related closely with SARS-CoV at the nucleic acid level despite the fact that they have over 72% sequence similarity. Moreover, SARS-CoV-2 strains from different regions were hard to solved completely based on genome sequences with multiple polytomies presented on the genome tree, particularly in the later time of this pandemic (Supplementary Fig. S1). We focused on the strains that are closer to the original direction and collected earlier in China. Since the branches on the basal of the tree have higher bootstrap values and divergent more than others (Fig. 1b), it may indicate that SARS-CoV-2 is slowly adapting to the human hosts’ environment in the course of COVID-19. The complete phylogenomic tree is shown in Supplementary Fig. S1.

**Figure 2:**
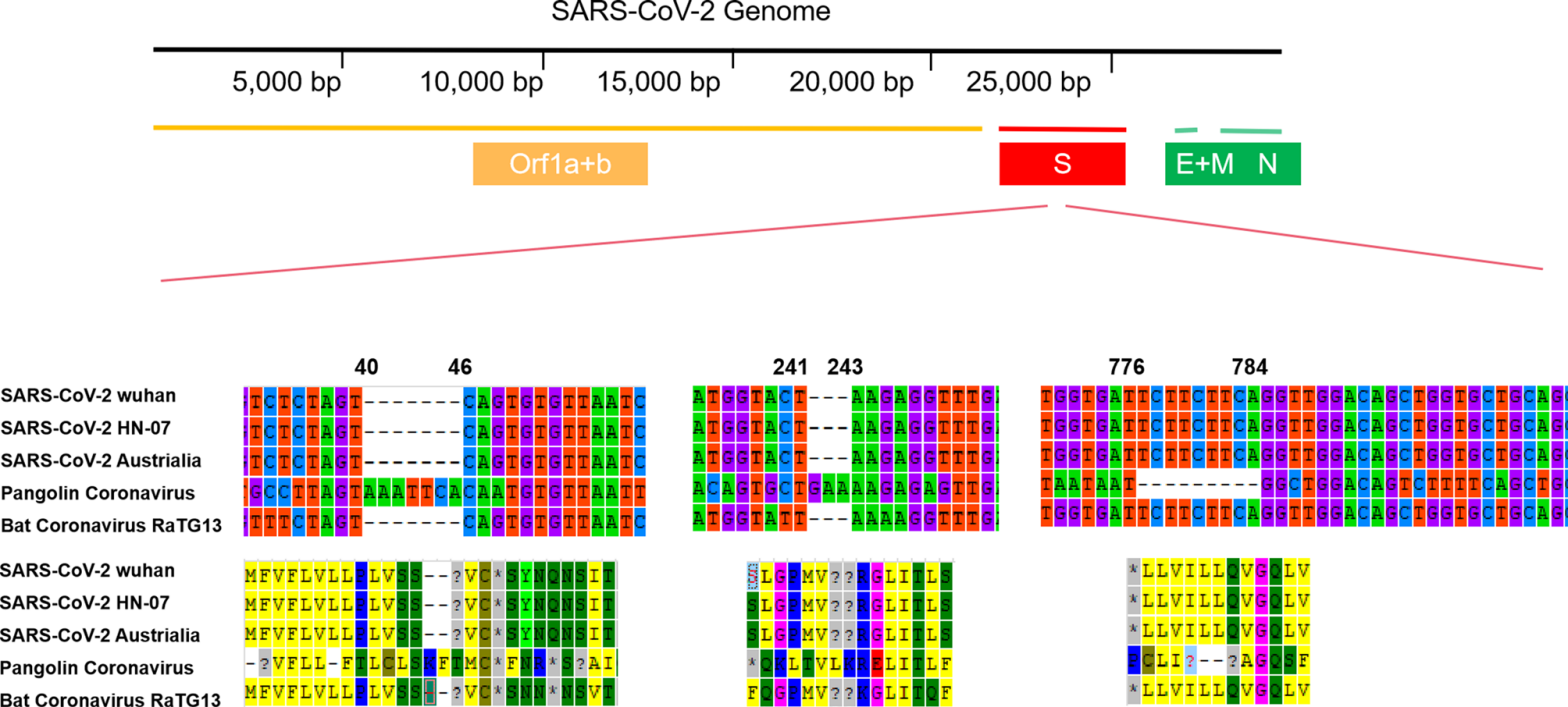
The sequence and amino acid alignment of the S gene in SARS-CoV-2 and coronaviruses found in bat and pangolin. The genomic structure of SARS-CoV-2 is shown in upper, the nucleic sequences alignment is shown in the middle while the amino acid is shown at the bottom of the figure.

**Figure 3:**
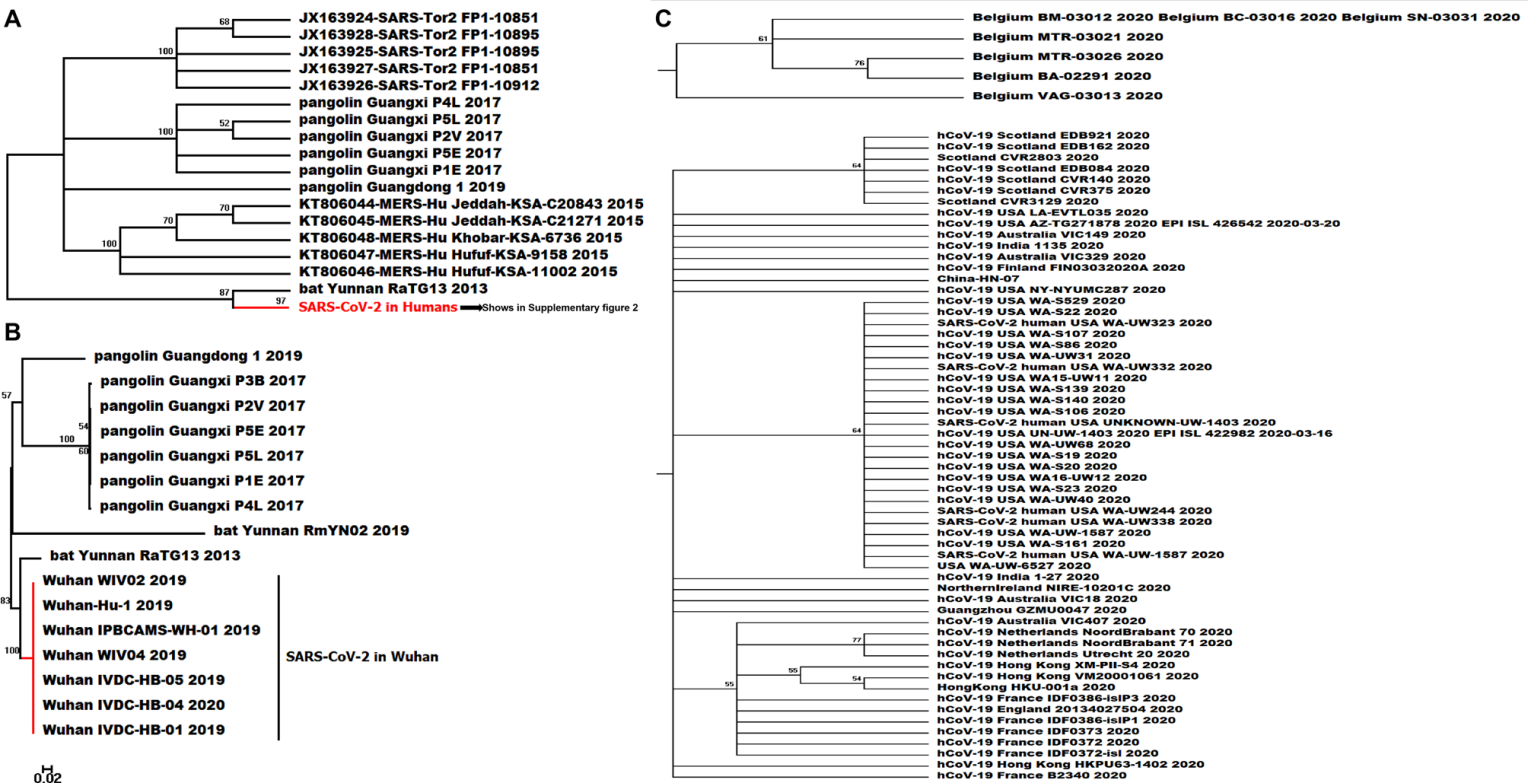
Phylogenetic tree based on S gene (Partial, the complete tree was found in Supplementary Fig. S2), the bootstrap values were mapped on the branch as long as the colors annotated for all clades (**A**) The phylogenetic relationship of SARS-CoV, MERS-CoV and coronaviruses in pangolin and bat, SARS-CoV-2 in human on the bottom includes branches of SARS-CoV-2 from 164 regions based on S sequences (Supplementary Fig. S2); (**B**) The phylogenetic relationship based on S gene sequences of SARS-CoV-2 collected from Wuhan city of China in the early time of COVID-19 pandemic and beta coronaviruses derived from bat and pangolin; (**C**) Clades of SARS-CoV-2 from the regions show more divergent in S gene along with the COVID-19 pandemic.

We extracted and aligned the S gene sequences of coronavirus from pangolin, bat (*Rhinolophus spp*) coronavirus RaTG13 and SARS-CoV-2 (Fig. 2). We found an insertion that caused frameshift in S genes of pangolin coronavirus, which demonstrated less similarity to the S gene in SARS-CoV-2 compared with that of RaTG13. However, the insertions exhibited in pangolin indicated potential recombination in the spike protein of coronavirus could occur during its cross-species transmission. According to S gene sequences in different strains, we found the similarity between SARS-CoV-2 and bat coronavirus RaTG13 is 98%, significantly higher than the similarity with pangolins (85%). S gene from different hosts showed higher divergence on the phylogenetic relationship (Fig. 3) compared with the genomic phylogenetic tree. Nevertheless, the phylogeny reconstructed based on genome, S gene, and multiple structural genes (S, E, M, N) all indicate the SARS-CoV-2 is more closely related to bat coronavirus than the coronaviruses isolated from pangolin.

The phylogenetic trees were also reconstructed based on the sequences of S, E, M, N genes (Supplementary Fig. S1), which were extracted from the genome sequences and SARS-CoV-2 Wuhan-01 was used as the reference genome. We believe those structural protein-coding genes are fundamental to viral infection^26^. Unlike the genomic phylogeny, structural protein-coding genes showed more divergence on two polar areas than the strains in the middle part of the tree. Similar to the genomic tree, multiple gene sequences of SARS-CoV-2 are divergent from outgroups and insertion or deletion were detected according to S gene sequences comparison.

### Polymorphism prediction of spike protein in SARS-CoV-2

**Figure 4:**
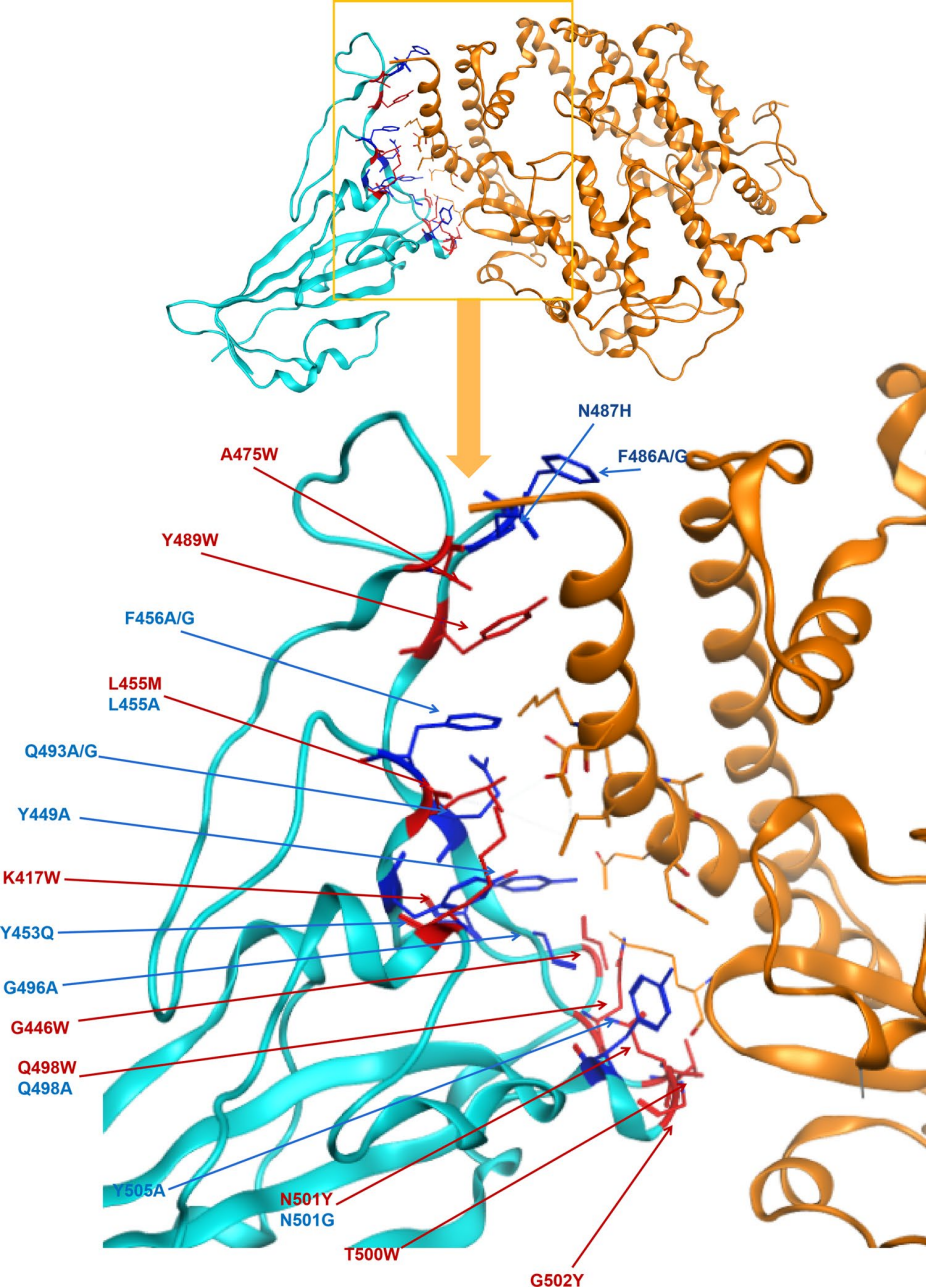
Identified variants in spike protein RBD mapped to the structure of spike protein in SARS-CoV-2 in complex with ACE2 in humans. Blue = spike protein, Orange = ACE2 direct binding to spike protein. Twenty-three point mutation causing affinity significant change on direct binding of spike protein of SARS-CoV-2, dark blue presents decreasing affinity while red shows increasing.

S gene has been studied as the key for SARS-CoV-2 binding to host cells’ receptors. This gene showed less conservation compared with the rest of the genome sequence of SARS-CoV-2. Some studies suggested that the high divergence in spike protein RBD and specifically the direct binding sites to the ACE2 receptor might play an important role in SARS-CoV-2′s adaptation to different animal hosts, and different human populations^16,27^. Combined with the phylogeny based on the S gene, we also simulated all possible missense mutations in the RBD of the S gene and evaluated their binding capacity with the ACE2 receptor of humans. Twenty-three sequence variants were predicted to show differential affinity and stability of the spike protein (Fig. 4), among which nine variants exhibited increased affinity and stability while 14 with decreased affinity and stability (Table 1) compared to the wild-type complex. All simulated outcomes of 3860 variants in spike protein are shown in Supplementary Table S2.

**Table 1:** The affinity and stability of different variants of spike protein RBD bound to hACE2.

Mutants	Affinity (kcal/mol)	dAffinity (kcal/mol)	Stability (kcal/mol)	dStability (kcal/mol)
K417W	− 90.17	− 3.68	− 3096.01	1.08
G446W	− 98.31	− 7.56	− 3100.18	− 0.42
Y449A	− 88.71	5.75	− 3098.95	2.41
Y453Q	− 85.63	3.36	− 3100.47	1.40
L455M	− 92.15	− 6.35	− 3102.42	1.05
F456A	− 80.25	4.66	− 3100.11	3.05
F456G	− 80.11	4.80	− 3099.85	3.31
A475W	− 101.02	− 7.18	− 3101.49	0.28
F486A	− 84.93	6.49	− 3099.87	1.99
F486G	− 84.72	6.70	− 3099.50	2.37
N487H	− 86.01	5.65	− 3098.24	0.79
Y489W	− 96.85	− 5.74	− 3101.93	0.35
Q493A	− 90.20	3.63	− 3099.00	1.21
Q493G	− 89.33	4.50	− 3098.68	1.52
G496A	− 85.87	4.21	− 3102.19	− 0.18
Q498A	− 83.68	3.42	− 3100.48	1.45
Q498W	− 92.51	− 5.41	− 3102.17	− 0.25
T500W	− 96.40	− 5.19	− 3101.31	0.27
N501G	− 85.81	3.33	− 3098.31	1.94
N501Y	− 92.70	− 3.55	− 3100.42	− 0.17
G502Y	− 95.82	− 6.55	− 3102.78	− 0.84
Y505A	− 82.39	5.33	− 3101.12	1.98

Figure 4 and Table 1 showed the missense mutations in spike protein RBD that have a strong affinity (Cutoff = 3) and stability (Cutoff = 1) with hACE2 receptors. Interestingly, residues L455, Q498, and N501 would have two potential mutations that leading to conflicting affinity changes, including increased affinity if leucine 455 changed to methionine, glutaurine 498 changed to tryptophan and asparagine 501 changed to tyrosine. At the same time, reduced affinity is predicted when leucine 455 changed to alanine, glutamine 498 changed to alanine, or asparagine 501 changed to glycine. The variants on the same residue causing opposite affinity fluctuation suggest the mutations on those amino acids may lead to distinct adaptation directions for SARS-CoV-2 to gain fitness in different hosts. Furthermore, Phe456, Gln493, and Phe486 carried two mutations that both result in affinity reduction. The stability of spike protein with different mutations was summarized in Table 1. In summary, we only found five mutants that could increase the stability of spike protein including G446W, G496A, Q498W, N501Y and G502Y, which was not consistent with the affinity.

**Figure 5:**
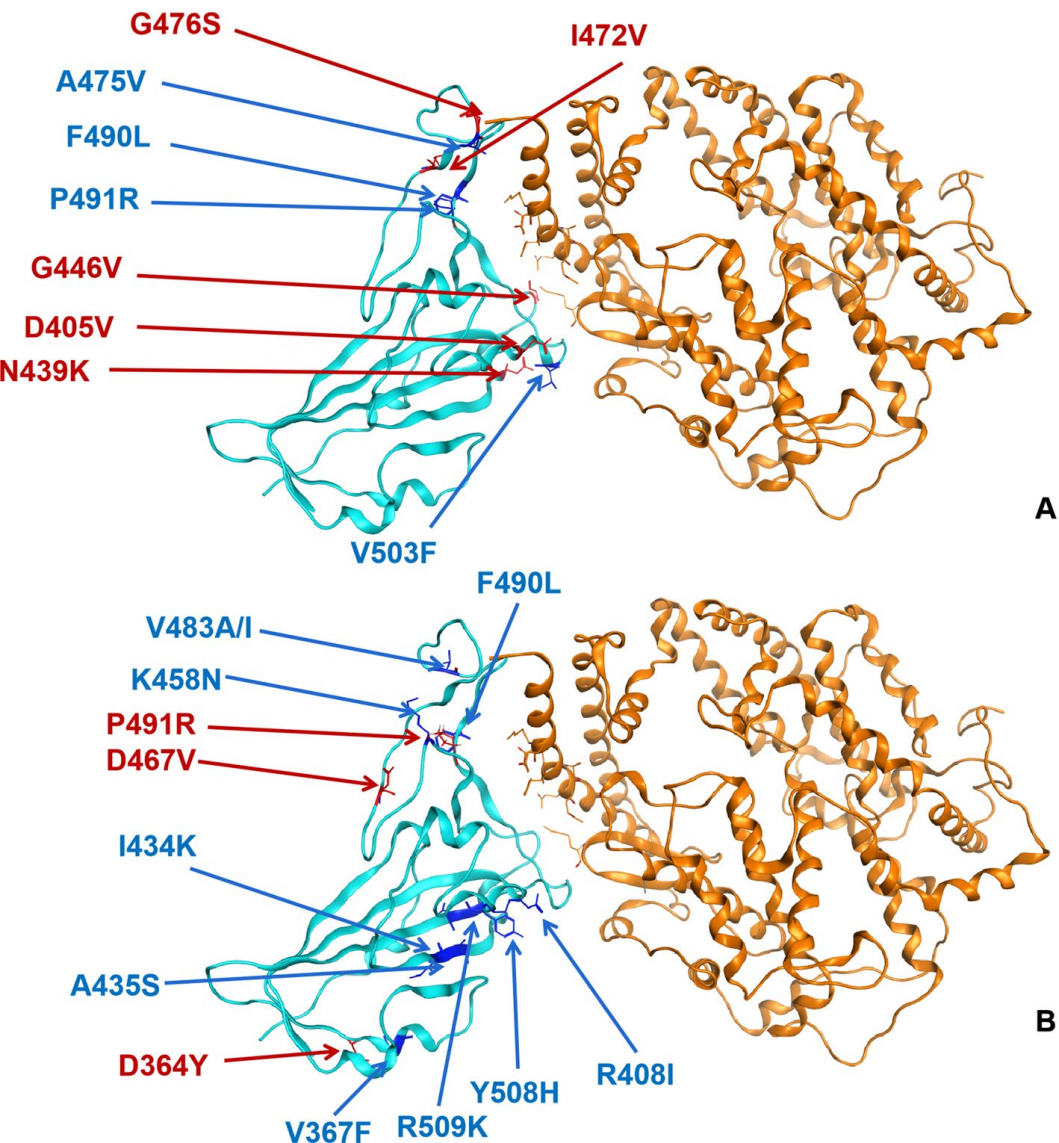
Reported variants in spike protein RBD mapped to the structure of spike protein in SARS-CoV-2 in complex with ACE2 in humans. Blue = spike protein, Orange = ACE2, dark blue presents decreasing affinity and stability while red presents increasing ones. (**A**) Affinity; (**B**) Stability.

We then investigated the mutations of spike protein reported by SARS-CoV-2 database of China National Center for Bioinformation (https://bigd.big.ac.cn/ncov) compared with our predictions. A total of 1,150 variants in spike protein were collected from the database and 643 missense variants (Before May 27th, 2020) were selected and analyzed their affinity and stability binding to hACE2 (Supplementary Table S3). Seventy-six missense variants in region T333-C525 of spike protein and nine variants showed changes in the affinity of it to ACE2 (Fig. 5A), and 13 variants cause structural stability change in spike protein RBD (Fig. 5B).

### Polymorphisms in ACE2 affect the binding ability to spike protein of SARS-CoV-2

We calculated the population frequency of 388 missense variants in ACE2 collected from both gnomAD (Supplementary Table S4) and the local population (Table 2). Analysis of their stability and affinity to spike protein of SARS-COV-2 were performed in this study, and the results showed no obvious trend of changes on affinity and stability found in all variants compared with the wild type hACE2 based on our cutoff of defining strong/weak affinity and stability.

**Table 2:** Analysis of affinity and stability of polymorphism in ACE2 from local population binding to spike protein of SARS-COV-2.

Mutants	Affinity (kcal/mol)	dAffinity (kcal/mol)	Stability (kcal/mol)	dStability (kcal/mol)
I468V	− 65.09	6.88E−12	− 3107.75	0.85
N638S	− 65.09	− 4.76E−12	− 5930.30	0.33
R708Q	− 64.99	0	− 2906.26	2.17

## Discussion and conclusions

Reconstructing the phylogeny of SARS-CoV-2 is of great value to understand its cross-species transmission pathway and to provide a reference for long-term infection prevention of zoonotic coronavirus. As a novel coronavirus, the morphological information of SARS-CoV-2 is limited for phylogenic analysis, while the genomic data provides a timely and accurate resource to identify its divergence during this pandemic of COVID-19^1,4^. The genomes of SARS-CoV-2 has been reported about ~ 80% similar to SARS-CoV^11^ and ~ 66% similar to MERS-CoV with low query coverage as 34%, but due to the structural and biological differences among them, they are strikingly different coronaviruses species^9,28^. The phylogeny of SARS-CoV-2 from over 50 countries in the present study shows both the genomic and S sequences of SARS-CoV-2 are more closely related to SARS-CoV compared with MERS-CoV. Despite the high genomic sequence similarity between SARS-CoV and SARS-CoV-2, the stability and viability of SARS-CoV-2 are obviously higher than SARS-CoV in both aerosol environments and objects surfaces^29^. This discrepancy indicates SARS-CoV-2 might adapt to human transmission better while SARS-CoV failed to do so^6,9,28,30^. Consistent with other studies^1,2,11,31^, we found all SARS-CoV-2 in our analysis were sister taxon with coronavirus from horseshoe bat. Bat coronavirus RaTG13 is highly suspected as the natural reservoir host of coronavirus^17,31^ and the similarity between SARS-CoV-2 and SARS-bat is as high as 96.12%. Meanwhile, the coronavirus isolated from pangolins were detected 91.02% identical with SARS-CoV-2 at the nucleotide level, which makes pangolins a potential intermediate host between the natural reservoir and human beings.

It is still uncertain that a common ancestral CoV that gave rise to SARS-CoV-2, bat coronavirus RaTG13 and pangolin coronavirus, but we found bat coronavirus RaTG13 is more closely related to SARS-CoV-2 in humans based on phylogenetic analysis of S gene. Furthermore, based on both genetic and genomic phylogeny, SARS-CoV-2 is highly likely to come from a bat coronavirus which has also been studied in some other analyses^1,31^. We found multiple insertions and deletions in S gene of SARS-pangolin compared with SARS-CoV-2 and SARS-bat, which suggests the pangolin might not be the only direct or intermediate host from cross-species transmission. Unique furin (polybasic) cleavage site insertion (PRRA) was found at the in-between region of S1 and S2 subunits of spike protein of SARS-CoV-2, and this structure has uncharacterized potentials to enhance the infectivity of SARS-CoV-2. Furthermore, this insertion has not been identified in SARS-pangolin and SARS-bat, which also indicates the essential intermediate host of SARS-CoV-2 remains unidentified^14,15,27^.

**Figure 6:**
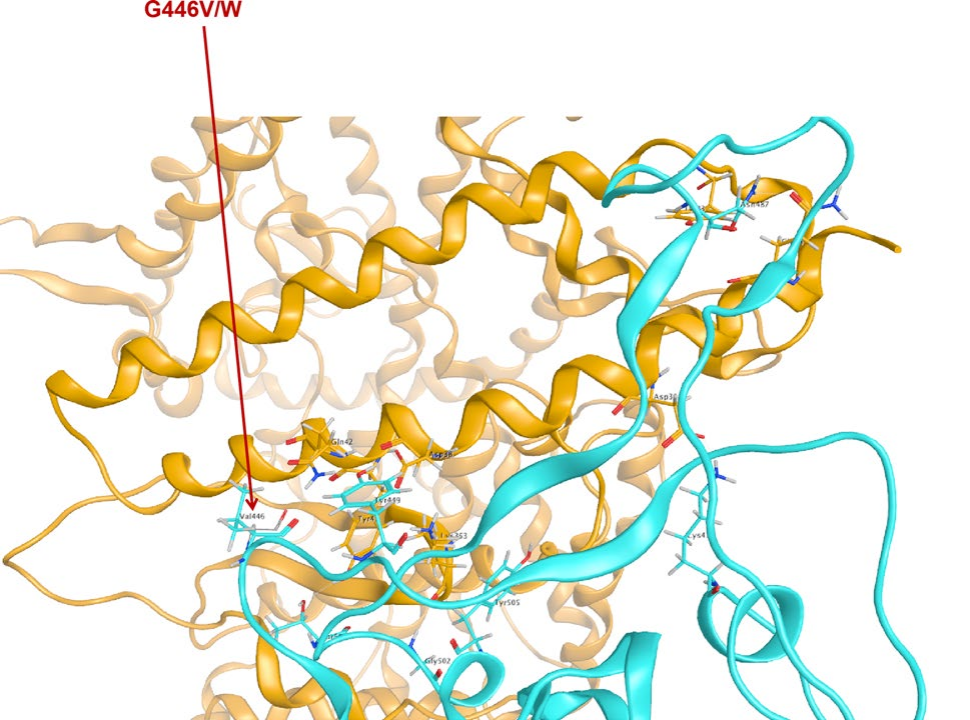
Residue G446 variants in documents from CDC (G446V) and in our prediction (G446W).

To most SARS-coronavirus, spike protein is the critical initiator for viral infection by recognizing the receptor of host cells^5,17^. S gene shows more divergence in sequences and protein structures relative to other regions of coronaviruse^14^. Along with gradual adaptation to the cellular environment of humans, mutations occur in spike protein, which might change the binding affinity and stability of the spike protein-hACE2 complex. An enhancement of such binding affinity and stability would increase the transmissibility of SARS-CoV-2 among humans and cause more severe disease^16^. For instance, the transmissibility and pathogenicity of SARS-CoV decreased comparing strains isolated in 2003–2004 with the ones from 2002 to 2003^32,33^. Similarly, MERS-CoV might have been controlled literally by itself along with spreading in human beings^9^. We predicted the affinity and stability of spike protein RBD (T333-C525) of SARS-CoV-2 by using MOE2019 (Molecular Operating Environment) to hACE2 (S19-D615), point mutations on spike protein were simulated with amino acid scan and 3860 mutations were set up for analysis (Supplementary Table S1). We selected 23 variants of spike protein RBD that causing significantly higher or lower stability and binding affinity to hACE2 with the cutoff set up as three for determining significance (Table 1). Nine mutations were found to significantly enhance the binding between spike protein and hACE2, which gives us an important direction in monitoring the arisal of mutations influencing the transmissibility of SARS-CoV-2 in the long run. Amino acid change on G446 (Fig. 6) was also reported through the pandemic from CDC (Fig. 6, Supplementary Table S2), and this sample was documented as collected in early March that is closer to the original direction of the phylogenetic tree based on genomic data (Fig. 1).

According to 1,150 variants in the spike protein of SASR-CoV-2 that including 634 missense mutations reported by CDC, we found 76 missense variants located in the region between T333-C525 and 5 variants enhanced its affinity binding to hACE2 (Table 3) and 3 of them increased the stability of spike-hACE2 complex (Table 4). Furthermore, we collected the genome sequences of SARS-CoV-2 harboring those variants in spike protein and found the variants V483A occured in 26 strains from the USA, V367F occured in 12 strains from Hong Kong, Australia, and other European countries, and a variant G446V with predicted highly enhanced affinity was found in a strain from Australia (Supplementary Table S2). We mapped those strains onto our genomic phylogenetic tree and found they dispersed distributed in the position close to the bat coronaviruses and pangolin coronaviruses. As COVID-19 spreads globally, few reports analyze the origin of SARS-CoV-2 according to the divergent pattern in spike protein. We found multiple variants in spike protein from different countries and clustered in the ancestral direction on phylogeny which might suggest the plasticity of spike protein is essential to the host-adaption of SARS-CoV-2 infection in humans and other hosts. However, due to the limited detection capability and restricted availability of samples from infected animals, the variants available in the national database are incompleted, more data is needed for identifying the patient zero.

**Table 3:** The affinity of the variants of SARS-CoV-2 spike RBD bound to human ACE2, red marks the enhanced ones while black represents the decreased ones.

Mutants	Affinity (kcal/mol)	dAffinity (kcal/mol)	Stability (kcal/mol)	dStability (kcal/mol)
D405V	− 88.38	− 0.35	− 791.79	0.24
N439K	− 88.46	− 0.46	− 796.46	0.57
I472V	− 87.92	− 0.80	− 799.30	0.99
G446V	− 91.90	− 1.15	− 3099.43	0.33
G476S	− 91.72	− 1.14	− 3100.24	0.63
A475V	− 90.61	3.24	− 3101.96	− 0.19
F490L	− 90.59	2.05	− 3100.21	1.28
P491R	− 88.25	2.31	− 803.62	− 0.59
V503F	− 91.94	1.54	− 3100.99	0.23

**Table 4:** The stability of the variants of SARS-CoV-2 spike RBD bound to human ACE2, red marks the enhanced ones while black represents the decreased ones.

Mutants	Affinity (kcal/mol)	dAffinity (kcal/mol)	Stability (kcal/mol)	dStability (kcal/mol)
D364Y	− 88.26	− 0.00	− 792.45	− 0.87
D467V	− 88.26	− 0.00	− 793.14	− 0.68
P491R	− 88.25	2.31	− 803.62	− 0.59
V367F	− 88.26	6.85E−12	− 799.28	1.06
R408I	− 88.26	− 0.00	− 792.67	1.44
I434K	− 88.26	1.27E−07	− 800.49	1.84
A435S	− 88.26	− 4.22E−05	− 799.69	1.04
K458N	− 88.186	0.06	− 793.07	1.29
V483A	− 88.606	− 0.012	− 797.29	1.01
V483I	− 88.726	− 0.13	− 797.21	1.09
F490L	− 90.596	2.05	− 3100.21	1.28
R509K	− 88.26	5.45E−05	− 797.38	2.04
Y508H	− 88.18	0.05	− 798.96	2.41

Given the SARS-CoV-2 strains have variants on spike protein relative to a reference sequence of SARS-CoV-2 (NC_045512.2) were found more divergence on the phylogenetic position that closer to coronaviruses isolated from bat and pangolin, it might indicate an evolutionary pattern occurred in S gene enabling SARS-CoV-2 adaptation to human hosts. However, since it is difficult to find the exact time the patient zero who got infected by SARS-CoV-2, the date of the sample collection could mislead the results. We believe further clinical information from all the countries would be needed and deeper cooperation and collaboration could be helpful. We shared all the analysis of over 3000 variants in spike protein to help the world tracking the mutations of SARS-CoV-2 and select the potential druggable targets or neutral inhibitors to prevent further life loss brought by the pandemic of COVID-19.

**Figure 7:**
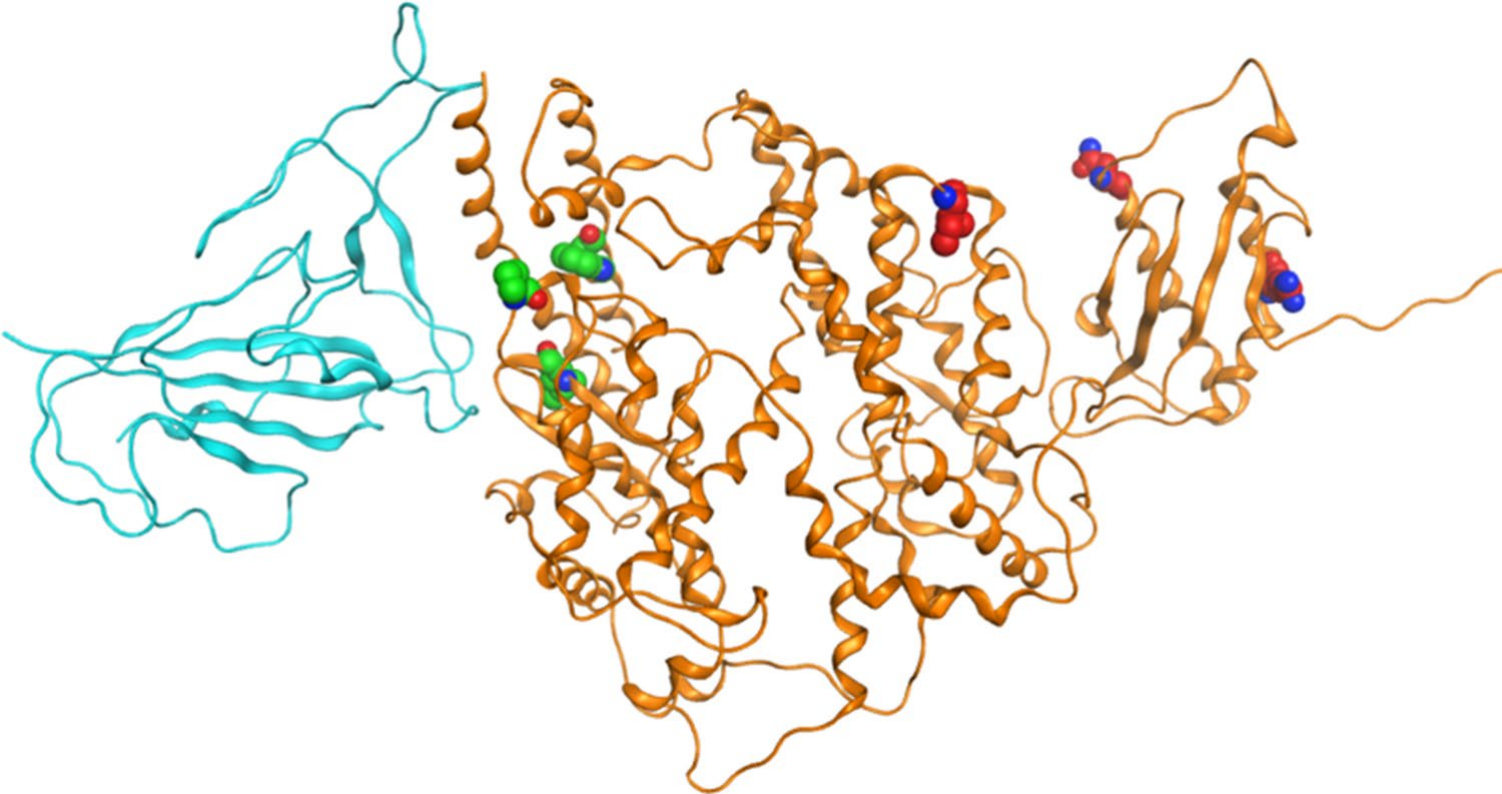
Spike protein polymorphous points from local population and Italy^37^, the Italian ones are marked as green while local ones marked as red.

Host-virus interaction over time makes a natural selection on both virus and host cells^34^. It is believed that the variants in hACE2 receptor play a role in SARS-CoV-2 infection. Cao and his colleagues (2020)^35^ found the polymorphisms of hACE2 did not bring differences in resisting SARS-CoV-2 infection, but the data they used is limited by sample size. A study published on bioRxiv showed hACE2 with the variants K26R, S16P, T27A, K31R, H34R, E35K, E37K, D38V, N51S, N64K, K68E, F72V, T921, Q102P, G326E, G352V, D355N, H378R, Q388L, and D509Y were able to increase the susceptibility of the individuals who carry these variations, while K31R, E35K, E37K, D38V, N33I, H34R, Q388L, and Y83H decreased binding capacity between hACE2 and spike protein of SARS-CoV-2. Herein, we identified all the variants in hACE2 in GnomAD and local dataset, with their population frequency in different ethnic groups calculated. Same with Eric’s results^36^, no significant differences in hACE2 variant frequency were found from gnomAD while three variants (I468V, N638S, and R708Q) with high allele frequency hACE2 were identified in the local population, and their allele frequencies were very low in other populations (Fig. 7). Researchers found three unique variants in hACE2 from the Italian population that might be corresponding to the high fatality of COVID-19 in Italy, which are P389H, W69C, and L351V^37^.

We compared the unique variants from the local and Italian populations by mapping them on the hACE protein structure (Fig. 7). The variants found in Italian were closer to the binding region of spike protein than Chinese variants; however, in silico simulation indicated that none of them change the affinity and stability of spike protein of SARS-CoV-2 and hACE2 complex significantly (Table 2). We did not find any variants in hACE2 that would increase or decrease the affinity and stability of spike protein binding to hACE2, and which may indicate SARS-CoV-2 enables indiscriminately infect to all humans ethnic groups. Due to various diets and health conditions, the efficiency and virulence of SARS-CoV-2 might be different, and the mortality rate is also more likely to be linked to the medical and health conditions of patients.

Up to date, there is no effective therapeutics approved universally for both treatment and prevention of COVID-19. To develop an efficient inhibitor or vaccine to prevent SARS-CoV-2 leading to COVID-19, it is still urgent to understand the mechanism of SARS-CoV-2 adaptation and transmission in different hosts, particularly in humans. The variants in the spike protein of SARS-CoV-2 and hACE2 would provide a database for tracking the adaptive mutation of SARS-CoV-2 and potential recombination events across different species. Our ongoing study shows ORF1ab of SARS-CoV-2 and MERS-CoV may undergo recombination and result in more severe disease (unpublished). The analysis shared in this study would provide useful genetic information to prevent the recurrence of this epidemic, and protect human beings from zoonotic coronavirus infection in the future.

## Supplementary information

Supplementary Information 1

Supplementary Information 2

Supplementary Information 3

Supplementary Information 4

Supplementary Information 5

Supplementary Information 6

Supplementary Information 7

Supplementary Information 8

Supplementary Information 9

Supplementary Information 10
