# Supplementary figures and images for: Dynamics of binding ability prediction between spike protein and human ACE2 reveals the adaptive strategy of SARS-CoV-2 in humans
Source: Sci Rep. 2021 Feb 4;11:3187. doi: 10.1038/s41598-021-82938-2 (PMC7862608; doi:10.1038/s41598-021-82938-2)

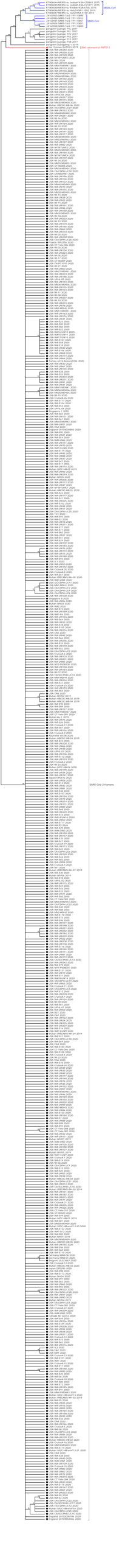

Supplement: Supplementary file 1 — Supplementary Information 1 [file 41598_2021_82938_MOESM1_ESM.pdf]

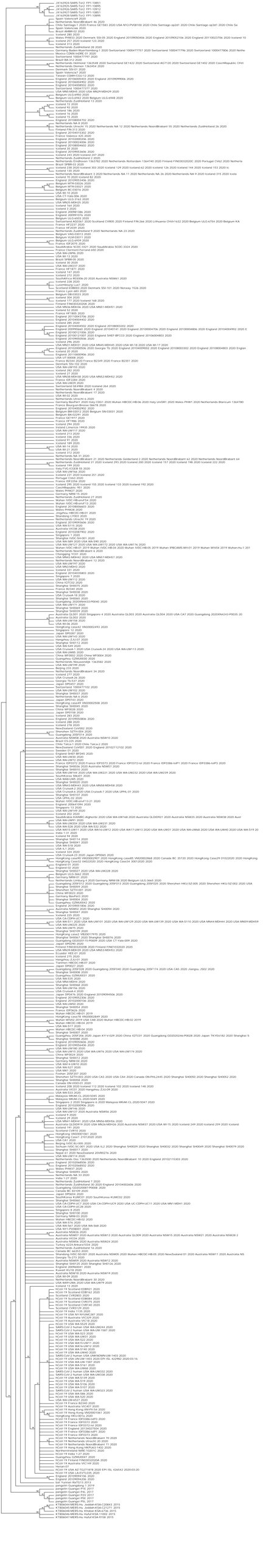

Supplement: Supplementary file 2 — Supplementary Information 2 [file 41598_2021_82938_MOESM2_ESM.pdf]

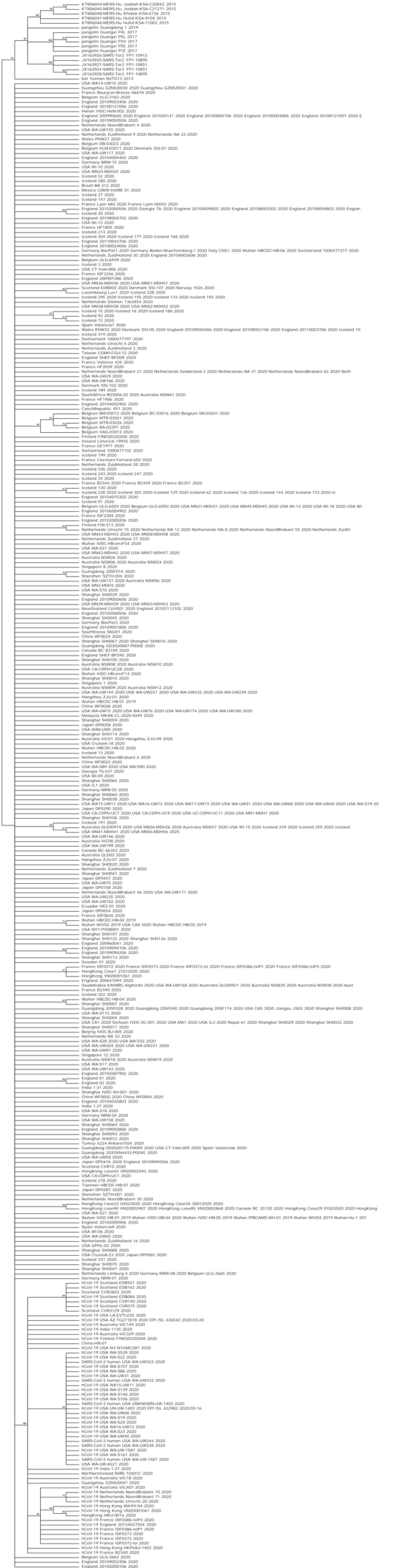

Supplement: Supplementary file 3 — Supplementary Information 3 [file 41598_2021_82938_MOESM3_ESM.pdf]
