# Supplementary material for: Dynamics of binding ability prediction between spike protein and human ACE2 reveals the adaptive strategy of SARS-CoV-2 in humans
Source: Sci Rep. 2021 Feb 4;11:3187. doi: 10.1038/s41598-021-82938-2 (PMC7862608; doi:10.1038/s41598-021-82938-2)
Supplement: Supplementary file 4 — Supplementary Information 4 [file 41598_2021_82938_MOESM4_ESM.pdf]

Dynamics of binding Ability Prediction between Spike Protein and Human ACE2 Reveals the Adaptive Strategy of SARS-CoV-2 in Humans

Supplementary Information

Xia Xue[1,3*], Jianxiang Shi[1,5*], Hongen Xu[1*], Yaping Qin[1,2], Zengguang Yang[1], Shuaisheng Feng[1], Danhua Liu[1,2], Liguo Jian[2], Linlin Hua[2], Yaohe Wang[3,4], Qi Zhang[6], Xueyong Huang[7], Xiaoju Zhang[8], Xinxin Li[9], Chunguang Chen[9], Jiancheng Guo[1,2,5#], Wenxue Tang[1,2,5#], Jianbo Liu[2#]

*Joint first authors
#Joint corresponding authors
1 Academy of Medical Sciences, Precision Medicine Center of The Second Affiliated Hospital of Zhengzhou University, Zhengzhou University
2 The Second Affiliated Hospital of Zhengzhou University
3 National Centre for International Research in Cell and Gene Therapy, Academy of Medical Science, Zhengzhou University
4 Center for Biomarkers & Biotherapeutics, Barts Cancer Institute, Queen Mary University of London
5 BGI College, Henan Institute of Medical and Pharmaceutical Sciences, Zhengzhou University, Henan, China
6 State Key Laboratory of Esophageal Cancer Prevention & Treatment; School of Pharmaceutical Sciences, Zhengzhou University
7 Henan Province Center for Disease Control and Prevention, Zhengzhou, China
8 Henan Provincial People's Hospital, China
9 Henan Hospital of Infectious Diseases, China

Contents

1 Supplementary figure 1 the ML phylogeny tree of different strains SARS-CoV-2
2 Supplementary figure 2 phylogenetic tree based on S gene
3 Supplementary figure 3 the ML phylogeny tree of different strains SARS-CoV-2 S, E, M, N
4 Supplementary table 1 metadata of SARS-CoV-2
5 Supplementary table 2 all analysis outcomes of 3,860 variants in spike protein
6 Supplementary table 3 all analysis outcomes of 1,150 variants in hACE
7 Supplementary table 4 the population frequency of 388 missense variants in ACE2
8 Supplementary script 1 the custom Python script was used to extract sequences
9 Supplementary script 2 The Perl script used for sequence filtering
