# Supplementary material for: Dynamics of binding ability prediction between spike protein and human ACE2 reveals the adaptive strategy of SARS-CoV-2 in humans
Source: Sci Rep. 2021 Feb 4;11:3187. doi: 10.1038/s41598-021-82938-2 (PMC7862608; doi:10.1038/s41598-021-82938-2)
Supplement: Supplementary file 5 — Supplementary Information 5 [file 41598_2021_82938_MOESM5_ESM.pdf]

```
import os,re,sys

my_fa_input = sys.argv[1]
my_gene_pos = sys.argv[2]      ### S              68373       79614
my_output = open(sys.argv[3],'w')


my_seq_dict = {}
with open(my_fa_input) as fh1:
    for line in fh1:
        line    = line.strip()
        if line.startswith(">"):
            header = line
        else:
            sequnce = line
            my_seq_dict[header] = sequnce

for h,seq in my_seq_dict.items():
    my_output.write(h+"\n")
    with open(my_gene_pos) as fh2:
        for line2 in fh2:
            line2 = line2.strip().split("\t")
            pos1 = int(line2[1])
            pos2 = int(line2[2])
            my_output.write("{}".format(seq[pos1-1:pos2-1]))
    my_output.write("\n")
```
