# Supplementary material for: Dynamics of binding ability prediction between spike protein and human ACE2 reveals the adaptive strategy of SARS-CoV-2 in humans
Source: Sci Rep. 2021 Feb 4;11:3187. doi: 10.1038/s41598-021-82938-2 (PMC7862608; doi:10.1038/s41598-021-82938-2)
Supplement: Supplementary file 6 — Supplementary Information 6 [file 41598_2021_82938_MOESM6_ESM.pdf]

```
#! /usr/bin/perl -w
use strict;
die "perl $0    <fa>\n" unless    @ARGV==2;
my ($fa,$out)=@ARGV;

$fa=~/gz$/?(open IN,"gzip -cd $fa|"||die):(open IN,$fa||die);
$/=">";<IN>;$/="\n";
my (%out,@list,%rmdup);
while(<IN>){
	my $info=$1 if(/^(.*)/);
	push @list,$info;
	$/=">";
	my $seq=<IN>;
#	print "$seq";
	$/="\n";
	$seq=~s/>|\r|\n//g;
	$seq=~s/-//g;
	next if $seq =~ /NNNNNNNNNNNNNNN/;
	if (not exists $rmdup{$seq}){
		$rmdup{$seq} = $info;
	} else {
		$rmdup{$seq} .= "__$info";
		next;
	}
#	print ">$info\n$seq\n";
#	$out{$info} = $seq;
#		print "x";
#	print ">$info\n$seq" if(exists $ha{$info} && ! exists $out{$info});
#	$out{$info}=1;
}
close IN;
my $n =0;
foreach my $k(keys %rmdup){
	print ">$rmdup{$k}\n$k\n";
}
#print "$n\n";
#foreach my $j(@list){
#	print ">$j\n$out{$j}\n";
#}
```
